# Adiponectin rs1501299 and chemerin rs17173608 gene polymorphism in children with type 1 diabetes mellitus: relation with macroangiopathy and peripheral artery disease

**DOI:** 10.1007/s40618-023-02215-z

**Published:** 2023-10-13

**Authors:** N. Y. Salah, S. S. Madkour, K. S. Ahmed, D. A. Abdelhakam, F. A. Abdullah, R. A. E. H. Mahmoud

**Affiliations:** 1 Pediatrics Department, Faculty of Medicine, Ain Shams University, Cairo, Egypt; 2 Radiology Department, Faculty of Medicine, Ain Shams University, Cairo, Egypt; 3 Clinical Pathology Department, Faculty of Medicine, Ain Shams University, Cairo, Egypt; 4 Department of Diagnostic and Interventional Radiology and Molecular Imaging, Ain Shams University, Cairo, Egypt

**Keywords:** Chemerin, Adiponectin, Polymorphism, Peripheral artery disease, Children, Type 1 diabetes

## Abstract

**Aim:**

Although macrovascular complications represent the leading cause of mortality in type 1 diabetes mellitus (T1DM), the prevalence of subtle macrovascular affection including peripheral artery disease (PAD) among children with T1DM and its genetic predictors remains to be unraveled. Increasing evidence suggests a link between adiponectin rs1501299 and chemerin rs17173608 gene polymorphism and atherogenesis, and insulin resistance. Hence, this study assess the prevalence of these variants among children with T1DM in comparison to healthy controls and their association with macrovascular complications, namely PAD and hyperlipidemia.

**Methods:**

Fifty children with T1DM and 50 matched controls underwent a thorough assessment including adiponectin rs1501299 and chemerin rs17173608 gene polymorphisms, fasting lipids, glycated hemoglobin (HbA1c), and ankle–brachial index (ABI). Cochran–Armitage trend test was used to decide the risk allele and evaluate the association between the candidate variant and PAD using a case–control design.

**Results:**

Children with T1DM were found to have significantly higher ABI (*p* = 0.011) than controls. Chemerin gene polymorphism was detected in 41 children with T1DM (82.0%), while adiponectin gene polymorphism was detected in 19 children (38.0%). Children with T1DM having GG chemerin variant and those having TT adiponectin variant had significantly higher cholesterol with significantly lower HDL-C and ABI than those having the other two variants (*p* < 0.005). Children with T1DM having abnormal ABI had significantly higher chemerin G (*p* = 0.017) and adiponectin T (*p* = 0.022) alleles than those with normal ABI. Cholesterol and ABI were independently associated with chemerin and adiponectin gene polymorphism by multivariable regression analysis.

**Conclusion:**

Children with T1DM having chemerin and adiponectin gene polymorphisms have significantly higher cholesterol and ABI than those without these polymorphisms and controls.

**Trial registration:**

The Research Ethics Committee of Ain Shams University, approval number R 31/2021.

## Introduction

Diabetes with its two major types, type 1 (T1DM) and type 2 (T2DM), has long been recognized as a major risk factor for the development of vascular disease [1]. In adults, macrovascular complications are the leading cause of diabetes-related morbidity and mortality, namely coronary, cerebrovascular, and peripheral arterial disease (PAD) [2]. However, in childhood T1DM, the role of macrovascular complications is underestimated with more focus on diabetic microvascular complications.

While macrovascular complications are rare until adulthood, early subtle macrovascular changes have been shown to present since childhood, making children with T1DM at risk of early adult-onset vascular disease [3]. Predictors of PAD include glycemic and lipid derangements, diabetes duration, hypertension, and male gender [4]. However, not all people with T1DM having poor glycemic and lipid control develop PAD. Moreover, aggressive glycemic control to lower the HbA1c did not appear to reduce the rate of PAD in those at risk as observed in the DCCT/EDIC study [5]. Hence, understanding the genetic determinants of the risk of PAD among this vulnerable population is of utmost importance.

Ankle–brachial index (ABI), i.e., the ratio of ankle-to-brachial systolic blood pressure, is the gold standard noninvasive method for PAD diagnosis [6]. According to the AHA, an ABI ≤ 0.90 is considered PAD. ABI can detect PAD, with > 50% stenosis with sensitivity 61–73% and specificity 83–96% [7].

Increasing evidence suggests a link between adipokines, diabetes, and vascular diseases including PAD [8]. Two of the most well-known adipokines, adiponectin and chemerin, have been associated with metabolic, inflammatory, and atherosclerotic diseases. Serum adiponectin levels have been associated with vascular events and/or mortality from vascular diseases [9]. Chemerin, an adipokine highly expressed in the white adipose tissue, is associated with inflammation and adipogenesis. Chemerin not only regulates the expression of adipocyte genes linked with glucose and lipid homeostasis, but also affects innate and adaptive immunity as well as cytokine homeostasis. Furthermore, it has a role in fibrinolysis, coagulation, and inflammation [10]. Hence, adipokines could serve as a mechanistic link between diabetes, metabolic derangements, and PAD. There remains uncertainty, however, about the strength of associations between PAD and individual adipokines with some studies suggesting that these associations are mediated through diabetes [11].

Adiponectin level is influenced by the presence of genetic variation in its coding genes. Polymorphisms in ADIPOQ, which codes for adiponectin, have been associated with diabetes and metabolic syndrome in multiple populations [12]. Nonetheless, genetic variation in the ADIPOQ promoter has been associated with vascular disease independent of adiponectin level [13].

Variants of retinoic acid receptor responder 2 (RARRES2), the gene encoding chemerin, have been demonstrated to be associated with increased chemerin levels, visceral fat mass in nonobese individuals, and increased incidence of metabolic syndrome [14]. Several experimental studies suggest an inflammatory role for chemerin during the early stages of vascular diseases [15]. But it is currently still not clear whether an association between chemerin gene polymorphism and PAD exists. Some case–control studies have shown that higher circulating chemerin levels are associated with clinical and subclinical vascular diseases [16, 17], whereas others did not confirm these findings [18, 19].

Hence, this study aims to assess the frequency of adiponectin and chemerin gene polymorphism among children with T1DM in comparison to healthy control and to investigate their potential association with macrovascular complications, namely PAD and hyperlipidemia among this vulnerable cohort.

## Methodology

### Study design

This cross-sectional controlled study included 50 children with T1DM and 50 age—and gender-matched healthy controls. Children with T1DM were recruited from the pediatric and adolescents diabetes unit, Ain Shams University, while the controls were recruited from the outpatient clinic during the period from December 2021 to May 2022. T1DM was defined according to the criteria of the International Society of Pediatric and Adolescent Diabetes [20]. Inclusion criteria for T1DM children were age 8–18 years and diabetes duration of ≥ 1 year. Exclusion criteria included patients with other types of diabetes, e.g., T2DM and maturity-onset diabetes of the youth (MODY), patients with obesity, hypertension, or cardiovascular diseases, and patients with comorbid disorders that might cause PAD, e.g., systemic lupus and autoimmune thyroiditis.

**Fig. 1:**
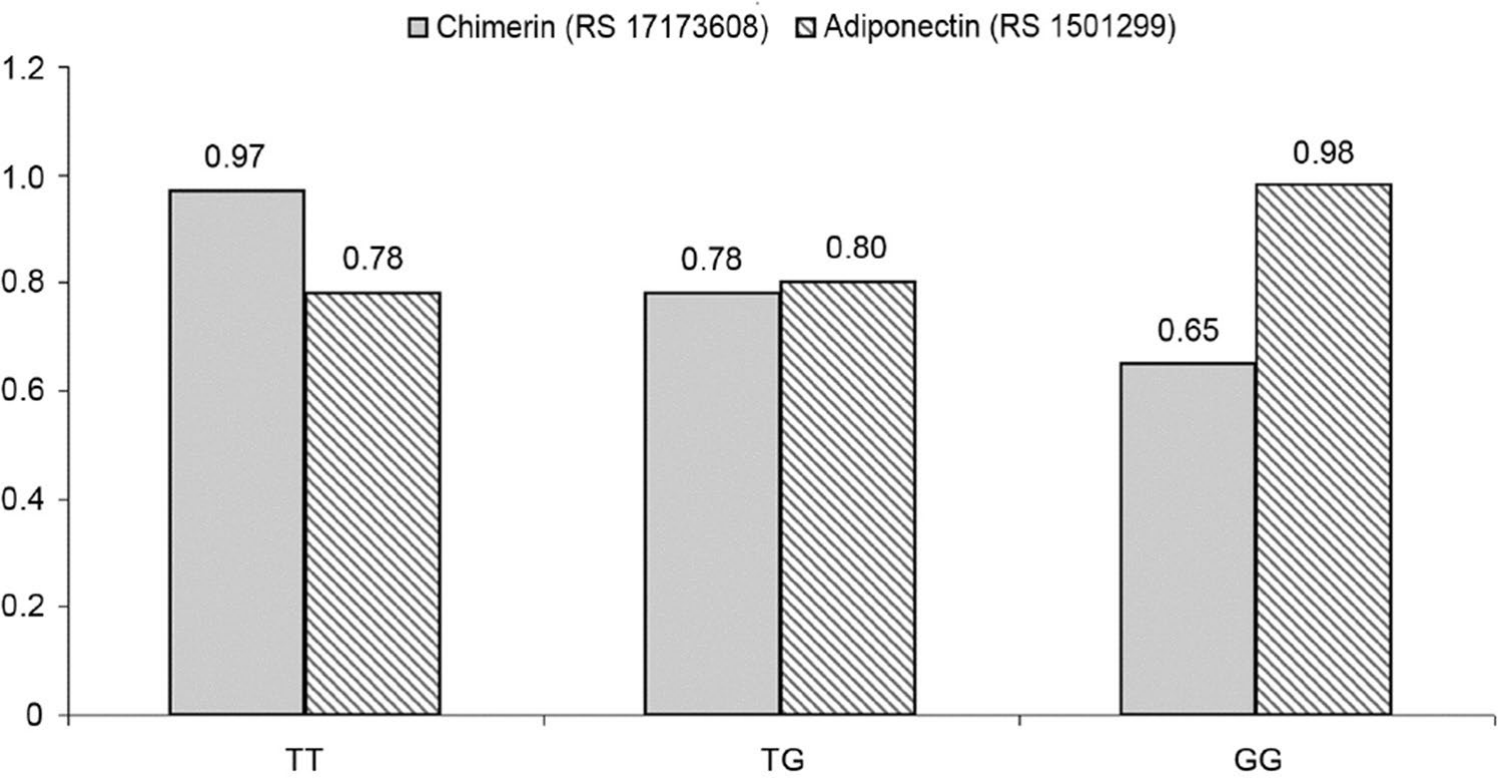
Relation of ABI to chemerin and adiponectin gene variants among the studied children with T1DM

### Ethical considerations

The study was approved by the Research Ethics Committee of Ain Shams University with an approval number of R 31/2021. Written informed consent was obtained from each subject and his legal guardian before enrollment in the study.

### Procedure

#### Clinical assessment

All included children were subjected to thorough medical history including patients' age, gender, family history of T1DM, diabetes duration, insulin therapy, and history of chronic microvascular diabetic complications (retinopathy, neuropathy, and nephropathy).

Clinical examination was done laying stress on anthropometric measures with the calculation of standard deviation score and body mass index (BMI) measured as kg/m^2^ [21].

Blood pressure (BP) was measured manually, two consecutive times, by a sphygmomanometer in the right arm of a relaxed, seated child with comparison of values to normal reference percentiles [22].

#### Laboratory assessment

##### Blood sampling

All blood samples were drawn after an overnight (10–12 h) fasting. Under complete aseptic condition, ten (10) mL of venous blood was withdrawn by venipuncture from each subject. Samples were divided into three aliquots: the first one was collected in vacutainer tubes (BD Diagnostics) containing tri-potassium ethylenediaminetetraacetate (k3 EDTA) and mixed gently and transferred immediately into appropriate containers in the laboratory, whereby DNA extraction was performed, and the DNA yields were stored at − 80 °C for genotyping.

The second aliquot was collected in plain vacutainer tubes for serum preparation. The serum is intended for measurement of lipid profile. The third aliquot was separated on ethylenediaminetetraacetic acid (1.2 mg/mL) for analysis of HbA1c.

##### DNA isolation and single-gene polymorphism genotyping

Genomic DNA was extracted from peripheral blood leukocytes using DNA purification mini-kit; QIAamp^®^ (Qiagen, Switzerland). All centrifugation steps for QIAamp mini spin columns were done at 6000 × *g* (8000 rpm) for 1 min at room temperature. The concentration and purity of the isolated DNA were checked using UV–vis absorption spectroscopy at *λ*260 nm by the Invitrogen Qubit^®^ 3.0 Fluorometer to measure the quantity of extracted DNA.

The genes chemerin [rs17173608 (T > G)] located in chromosome 7:150339575 (GRCh38.p13) within the noncoding regions and adiponectin [rs1501299 (G > T)] located in chromosome 3:186853334 (GRCh38.p13) within the adiponectin gene intron variant listed in the National Center for Biotechnology Information SNP database (http://www.ncbi.nlm.nih.gov/SNP) [23] were analyzed by RT-PCR using TaqMan^®^ genotyping master mix (Applied Biosystems, USA).

Genotyping of chemerin rs17173608 and adiponectin rs1501299 variant was performed using TaqMan allelic discrimination assay as recommended by Applied Biosystems International (ABI) [24], using the readymade genotyping assay kit supplied by ThermoFisher^®^ (ThermoFisher Scientific, USA) containing sequence-specific forward and reverse primers and two fluorescents (VIC/FAM) labeled TaqMan probes for distinguishing between the two alleles. The presence to the absence of the SNP and the allelic discrimination was established according to the type of the emitted fluorescence of either of the reporter dyes or both at the same time.

The reaction plates were loaded into the DT-lite Real-Time PCR System and amplification was performed according to the following protocol: initial heat activation at 95 °C for 10 min, DNA denaturation at 95 °C for 15s, followed by annealing at 60 °C for 60s for 40 cycles.

The data were analyzed and presented on the multicomponent plot for allelic discrimination of alleles.

1 versus Allele.

2 using Applied.

Biosystem, step I version software analysis modules.

##### Biochemical assessment

Fasting triglycerides, high-density lipoproteins (HDL-C), and total cholesterol were measured by standard automated methods on AU680 Beckman Coulter autoanalyzer (Beckman Coulter, Inc., Brea, CA) at Central Laboratories at Ain Shams Hospitals. LDL-C was estimated by Friedewald equation. Results were clinically interpreted according to recommendations of the European Atherosclerosis Society [25]. HbA1c was assessed using D-10 (Bio-Rad, France) [26].

#### Radiological assessment

The ankle–brachial index was assessed by two blinded experienced radiologists who assessed the patients separately and the mean score of both was taken for each subject. The subject was instructed to lie in the supine position. For measuring the brachial pressure, a fitting sphygmomanometer cuff was placed on the arm, with the upper limb at the same level as the heart. The ultrasound probe was put on the patient's antecubital fossa over the site of brachial artery pulsation. The cuff was inflated to about 10–20 mmHg above the expected systolic blood pressure of the patient till the duplex color and pulsed wave signal disappeared. Then slowly the cuff was deflated at approximately 1 mmHg/s. The brachial systolic pressure is the pressure when the duplex color and pulse wave signal re-appeared.

For assessing the ankle pressure, the sphygmomanometer cuff was placed just proximal to the malleoli. The ultrasound probe was placed slightly lateral to the midline of the dorsum of the foot to detect the dorsalis pedis color flow and pulse wave signal to measure the systolic blood pressure in the same steps done in the brachial artery systolic blood pressure measurement. Then the ultrasound probe was located posterior to the medial malleolus to detect the color flow and pulse wave signal of the posterior tibial artery in the foot. Both measurements were recorded on both legs.

The ABI was calculated for each leg separately by taking the higher pressure of the dorsalis pedis artery and posterior tibial artery and dividing it by the highest brachial arterial systolic pressure in both arms. Then the mean ABI was calculated for each subject [27].

### Statistical analysis

Data were collected, revised, coded, and entered into the Statistical Package for Social Science (IBM SPSS) version 25. The Kolmogorov–Smirnov test was used to examine the normality of data. Quantitative data were presented as mean, standard deviations, and ranges when their distribution was found parametric and median with inter-quartile range (IQR) when their distribution was found non-parametric. Qualitative data were presented as numbers and percentages. The one-way ANOVA test was used to compare parametric data sets, whereas non-parametric variables were compared using the Mann–Whitney test. Qualitative variables were compared using Chi-square (*X*^2^) test. To validate that genotype distribution follows Hardy–Weinberg equilibrium (HWE) model, goodness-of-fit Chi-square test was used. A Cochran–Armitage trend test was used to decide the risk allele and evaluate the association between the candidate variant and PAD using a case–control design [28]. Before multiple linear regression analysis, several variables were log-transformed to obtain an approximate normal distribution. Simple regression analysis was first performed to screen potential associations, followed by a multivariate stepwise linear regression model to identify and determine significant associations for chemerin and adiponectin gene polymorphism. Using the approach of stepwise variable selection, stepping up, only variables with a significance level of 0.05 were included in the model. Spearman correlation coefficients were used to assess the correlation between two quantitative parameters in the same group. The confidence interval was set to 95% and the margin of error accepted was set to 5%. So, the *p* value was considered significant at the level of < 0.05.

## Results

Fifty children with T1DM (26 males and 24 females; ratio 1.08:1) were studied. Their mean age was 11.09 ± 2.26 years, range 8–16, and their mean HbA1c was 9.72 ± 2.03%, range 6.6–13.7. They were compared to 50 age—and gender-matched healthy controls (*p* = 0.07 and *p* = 0.42, respectively). The clinical, laboratory, and radiological data of the studied children with T1DM and controls are listed in Table 1.

**Table 1:** Clinico-laboratory and radiological characteristics of the studied children with T1DM and controls

		Children with T1DM	Controls	*P* value
		No. = 50	No. = 50	
*Clinico-demographic data*				
Age (years)	Mean ± SD	11.09 ± 2.26	10.22 ± 2.47	0.070•
	Range	8–16	6.08 ± 13.07	
Gender	Female	24 (48.0%)	20 (40.0%)	0.420*
	Male	26 (52.0%)	30 (60.0%)	
Weight Z-score	Median(IQR)	− 0.75 (− 1.09 to − 0.11)	− 0.99 (− 1.12 to − 0.68)	0.076^‡^
	Range	− 1.78 to 0.56	− 1.2 to − 0.27	
Height Z-score	Median(IQR)	− 0.59 (− 1.16 to 0.55)	− 0.8 (− 1.54 to − 0.13)	0.055^‡^
	Range	− 2.39 to 1.72	− 1.73 to 0.73	
BMI (kg/m^2^)	Mean ± SD	16.95 ± 1.85	16.39 ± 1.37	0.091**•**
	Range	13.85–20.93	13.85–19.76	
BMI Z-score	Median(IQR)	− 0.79 (− 0.86 to − 0.58)	− 0.79 (− 0.85 to − 0.72)	0.403^‡^
	Range	− 1.03 to − 0.31	− 1.03 to − 0.43	
Systolic blood pressure percentile	Mean ± SD	60.40 ± 17.72	55.60 ± 14.02	0.136**•**
	Range	50–90	50–90	
Diastolic blood pressure percentile	Mean ± SD	57.20 ± 15.52	55.60 ± 14.02	0.590**•**
	Range	50–90	50–90	
*Laboratory data*				
Cholesterol (mg/dl)	Mean ± SD	179.31 ± 50.58	153.12 ± 19.52	**0.001•**
	Range	35–285	123–179	
HDL (mg/dl)	Mean ± SD	46.60 ± 11.49	58.28 ± 5.56	** < 0.001•**
	Range	30–70	48–68	
LDL (mg/dl)	Mean ± SD	109.30 ± 40.12	101.42 ± 18.96	0.212**•**
	Range	22–201	69–129	
Triglycerides (mg/dl)	Mean ± SD	115.49 ± 94.86	86.26 ± 16.16	**0.034•**
	Range	25–503	48–114	
HbA1C%	Mean ± SD	9.72 ± 2.03	4.49 ± 0.50	** < 0.001•**
	Range	6.6–13.7	3.7–5.3	
Chimerin (RS17173608)	TT	3 (6.0%)	12 (24.0%)	0.041*
	TG	6 (12.0%)	5 (10.0%)	
	GG	41 (82.0%)	33 (66.0%)	
	T/G	0.12/0.88	0.29/0.71	**0.003**
Adiponectin (RS1501299)	GG	21 (42.0%)	31 (62.0%)	0.011*
	GT	10 (20.0%)	13 (26.0%)	
	TT	19 (38.0%)	6 (12.0%)	
	G/T	0.52/0.48	0.75/0.25	**0.001**
*Radiological data*				
Ankle–brachial index	Mean ± SD	0.86 ± 0.26	1.05 ± 0.13	**0.011•**
	Range	0.5–1.5	0.92–1.17	

Bold values indicates significant values

*T1DM* type 1 diabetes mellitus; *BMI* body mass index; *HbA1C* glycated hemoglobin; *LDL* low-density lipoproteins; *HDL* high-density lipoproteins

*Chi-square test

•Independent t test

^‡^Mann–Whitney test; *p* value < 0.05: significant

### Peripheral artery disease among children with T1DM

The mean ankle–brachial index of the studied children with T1DM was 0.86 ± 0.26; range 0.5–1.5, while that of the controls was 1.05 ± 0.13; range 0.92–1.17. Children with T1DM had significantly lower ABI than controls (*p* = 0.011).

Although ABI was significantly correlated to diabetes duration (*r* = 0.450), insulin dose (*r* = 0.318), cholesterol (*r* = 0.336), LDL (*r* = 0.441) and negatively correlated to HDL (*r* = − 0.280) among the studied children with T1DM, it was not correlated to HbA1C (*r* = 0.129), Table 2.

**Table 2:** Correlation of ankle–brachial index with various clinico-laboratory parameters among the studied children with T1DM

	Ankle–brachial index	
	*r*	*P* value
Age (years)	− 0.188	0.192
Diabetes duration (years)	0.450**	**0.001**
Weight z-score	0.199	0.165
Height z-score	0.324*	**0.022**
BMI z-score	0.064	0.660
Insulin dosage (IU/kg/day)	0.318*	**0.024**
Cholesterol (mg/dl)	0.336*	**0.017**
HDL (mg/dl)	− 0.280*	**0.049**
LDL (mg/dl)	0.441**	**0.001**
Triglycerides (mg/dl)	0.005	0.975
HbA1C%	0.129	0.374

Bold values indicates significant values

*T1DM* type 1 diabetes mellitus; *BMI* body mass index; *HbA1C* glycated hemoglobin; *LDL* low-density lipoproteins; *HDL* high-density lipoproteins

Spearman correlation coefficients, *P* < 0.05: significant

### Chemerin and adiponectin gene polymorphism among children with T1DM

Regarding chemerin and adiponectin gene polymorphisms, GG (mutant) chemerin genotype was detected in 41 children with T1DM (82.0%), while TT (mutant) adiponectin genotype was detected among 19 children with T1DM (38.0%).

Children with T1DM had significantly higher GG (mutant) chemerin polymorphism (*p* = 0.041) and TT (mutant) adiponectin polymorphism (*p* = 0.011) than controls, Table 1.

Upon assessing the relation of chemerin and adiponectin gene variants and clinico-laboratory and radiological parameters among the studied children with T1DM, those having GG chemerin variant and those having TT adiponectin variant had significantly higher cholesterol (*p* < 0.001 and *p* < 0.001) with significantly lower HDL-C (*p* = 0.012 and *p* = 0.023) and ABI (*p* = 0.017 and *p* = 0.035) than those having the other two variants; Table 3.

**Table 3:** Distribution of different chimerin and adiponectin gene variants in relation to the clinico-laboratory and radiological parameters among the studied children with T1DM

		Chimerin (RS17173608)			Test value	P value	Adiponectin (RS1501299)			Test value	*P* value
		TT	TG	GG			TT	TG	GG		
		No. = 3	No. = 6	No. = 41			No. = 19	No. = 10	No. = 21		
Diabetes duration (years)	Median (IQR)	6 (5–7)	7.5 (5–9)	7 (5–9)	0.481**‡**	0.786	7 (5–10)	7 (5–8)	6 (5–8)	1.523**‡**	0.467
	Range	5–7	4–12	2–13			2–13	2–12	2–13		
BMI z score	Median (IQR)	− 0.48 (− 0.58–− 0.43)	-0.34 (-0.43–-0.31)	-0.32 (-0.61–0.02)	1.007**‡**	0.604	− 0.27 (− 0.50–0.10)	− 0.44 (− 0.60–− 0.18)	− 0.37 (− 0.61–− 0.18)	2.428**‡**	0.297
	Range	− 0.58–− 0.43	− 0.45–-− 0.18	− 0.87–1.37			− 0.87–4.26	− 0.79–1.33	− 0.86–4.37		
Nephropathy		0 (0.0%)	1 (16.7%)	8 (19.5%)	0.729*	0.694	4 (21.1%)	1 (10.0%)	4 (19.0%)	0.569*	0.752
Neuropathy		0 (0.0%)	0 (0.0%)	4 (9.8%)	0.954*	0.621	2 (10.5%)	1 (10.0%)	1 (4.8%)	0.518*	0.772
Cholesterol (mg/dl)	Mean ± SD	96.33 ± 53.35	128.33 ± 28.44	192.84 ± 42.39	**12.598•**	** < 0.001**	213.00 ± 50.97	152.10 ± 30.15	161.79 ± 41.83	**9.253**•	** < 0.001**
	Range	35–132	78–156	138–285			132–285	78–181	35–228		
HDL (mg/dl)	Mean ± SD	51.67 ± 16.50	58.50 ± 6.02	44.49 ± 10.77	**4.859•**	**0.012**	40.99 ± 9.10	50.20 ± 14.53	49.95 ± 10.31	**4.101**•	**0.023**
	Range	35–68	50–65	30–70			30–60	30–70	35–68		
LDL (mg/dl)	Mean ± SD	86.33 ± 10.60	79.67 ± 38.40	115.32 ± 39.67	2.777•	0.072	117.79 ± 36.77	91.60 ± 27.88	110.05 ± 46.38	1.427•	0.250
	Range	75–96	30–130	22–201			52–201	30–130	22–201		
Triglycerides (mg/dl)	Mean ± SD	58.33 ± 30.57	78.50 ± 32.56	125.08 ± 101.48	1.221•	0.304	149.00 ± 135.07	88.84 ± 39.50	97.86 ± 56.34	2.024•	0.143
	Range	29–90	25–124	29–503			56–503	29–153	25–196		
HbA1C %	Mean ± SD	9.20 ± 2.51	8.97 ± 2.40	9.87 ± 1.97	0.612•	0.546	9.73 ± 1.83	10.00 ± 1.81	9.58 ± 2.35	0.140•	0.870
	Range	6.8–11.8	7–12	6.6–13.7			7–12.9	7–12	6.6–13.7		
Ankle–brachial index	Mean ± SD	0.97 ± 0.22	0.78 ± 0.23	0.65 ± 0.22	**4.456•**	**0.017**	0.78 ± 0.24	0.80 ± 0.14	0.98 ± 0.29	**3.607**•	**0.035**
	Range	0.5–1.5	0.53–0.99	0.55–0.98			0.5–1.49	0.53–0.98	0.65–1.5		

Bold values indicates significant values

*T1DM* type 1 diabetes mellitus; *BMI* body mass index; *HbA1C* glycated hemoglobin; *LDL* low-density lipoproteins; *HDL* high-density lipoproteins

•On–way ANOVA

^‡^Kruskal–Wallis test

*Chi-square test; *p* value < 0.05: significant

### Chemerin and adiponectin gene polymorphism in relation to peripheral artery disease

As shown in Fig. 1 and Table 4, the G chemerin allele and the T adiponectin allele were found to be significantly associated with abnormal ABI (*p* = 0.017 and *p* = 0.022, respectively). Moreover, the chemerin TT polymorphism was found to be significantly related to ABI in the co-dominant and recessive models (*p* = 0.017 and *p* = 0.030), while the adiponectin TT polymorphism was found to be significantly associated with abnormal ABI in the co-dominant model (*p* = 0.044).

**Table 4:** Associations of chimerin and adiponectin gene polymorphism with the risk of PAD among children with T1DM

Adiponectin (RS1501299)	Abnormal ABI	Normal ABI	OR (95% CI)	*P* value	OR (95% CI)*	*P* value	*Chimerin* (RS17173608)	Abnormal ABI	Normal ABI	OR (95% CI)	*P* value	OR (95% CI)*	*P* value
	No. = 14	No. = 36						No. = 14	No. = 36				
Codominant													
TT	8 (57.1%)	11 (30.6%)	Ref		Ref		TT	7 (50.0%)	34 (94.4%)	Ref		Ref	
GT	3 (21.4%)	6 (16.7%)	0.217 (0.047–0.993)	**0.049**	0.198 (0.041–0.956)	**0.044**	TG	5 (35.7%)	1 (2.8%)	0.103 (0.008–1.298)	0.079	0.288 (0.018–4.511)	0.375
GG	3 (21.4%)	19 (52.8%)	0.316 (0.05–1.998)	0.221	0.343 (0.052–2.267)	0.267	GG	2 (14.3%)	1 (2.8%)	0.041 (0.004–0.409)	**0.006**	0.053 (0.005–0.591)	**0.017**
Recessive													
TT	8 (57.1%)	11 (30.6%)	Ref		Ref		GG	2 (14.3%)	1 (2.8%)	Ref		Ref	
GT + GG	6 (42.9%)	25 (69.4%)	3.03 (0.848–10.835)	0.088	3.461 (0.898–13.337)	0.071	TG + TT	12 (85.7%)	35 (97.2%)	5.833 (0.484–70.244)	0.165	2.371 (0.159–35.335)	0.531
Dominant													
TG + TT	11 (78.6%)	17 (47.2%)	Ref		Ref		TG + GG	7 (50.0%)	3 (8.3%)	Ref		Ref	
GG	3 (21.4%)	19 (52.8%)	4.098 (0.976—17.202)	0.054	4.211 (0.973—18.231)	0.055	TT	7 (50.0%)	33 (91.7%)	11.000 (2.267–53.372)	**0.003**	6.310 (1.195—33.329)	**0.030**
Allele													
T	19 (67.9%)	28 (38.9%)	Ref		Ref		T	19 (67.9%)	69 (95.8%)	Ref		Ref	
G	9 (32.1%)	44 (61.1%)	3.317 (1.317–8.357)	**0.011**	3.300 (1.191–9.140)	**0.022**	G	9 (32.1%)	3 (4.2%)	10.895 (2.682–44.262)	**0.001**	5.995 (1.375–26.136)	**0.017**

Bold values indicates significant values

*Adjusted for age and gender, *p* value < 0.05: significant

Upon performing univariate and multivariate regression analyses for factors associated with chemerin and adiponectin gene polymorphism among children with T1DM, cholesterol and ABI were the significant independent variables related to chemerin (*p* = 0.03 and *p* = 0.038, respectively); as for adiponectin gene polymorphism, it was independently related to cholesterol, triglycerides, and ABI (*p* = 0.016, *p* = 0.023, and *p* = 0.021), Table 5.

**Table 5:** Univariate and multivariate logistic regression analysis (using backward Wald method) for factors associated with chimerin GG polymorphism and adiponectin TT polymorphism among children with T1DM

	Chimerin				Adiponectin			
	Univariate		Multivariate		Univariate		Multivariate	
	OR (95% C.I.)	*P* value	OR (95% C.I.)	*P* value	OR (95% C.I.)	*P* value	OR (95% C.I.)	*P* value
Cholesterol (mg/dl)	**1.143 (1.038–1.259)**	**0.007**	**1.212 (1.019–1.440)**	**0.030**	1.032 (1.012–1.053)	**0.002**	1.028 (1.005–1.052**)**	**0.016**
HDL (mg/dl)	**0.907 (0.842–0.978)**	**0.011**			0.921 (0.864–0.981)	**0.011**	–	–
LDL (mg/dl)	**1.027 (1.003–1.052)**	**0.031**			1.007 (0.999–1.016)	0.093	–	–
Triglycerides (mg/dl)	1.017 (0.997–1.038)	0.094			23.388 (1.712–319.465)	**0.018**	**17.541 **(**9.053–44.911)**	**0.023**
Ankle–brachial index	**11.000 (2.267–53.372)**	**0.003**		**6.512 (1.177–31.418)**	**0.038**	**16.250 (1.917–137.778)**	**0.011**	**13.232 (1.839–127.572)**

Bold values indicates significant values

*T1DM* type 1 diabetes mellitus; *LDL* low-density lipoproteins; *HDL* high-density lipoproteins

*P* value < 0.05: significant

## Discussion

Subclinical PAD detected as increased ABI has been documented among youth with T1DM. This subclinical vascular affection is recognized as a strong independent predictor for future cardiovascular and cerebrovascular morbidity and mortality [29, 30]. PAD is a complex dynamic process modified by a variety of molecular signaling pathways and genetic determinants [31]. An imbalance between pro-inflammatory and anti-inflammatory cytokines is among the suggested pathways.

Polymorphism of the genes encoding these adipokines may play a role in this process. Hence, assessing the role of these adipokines polymorphisms in the occurrence of PAD among children with T1DM is of utmost important aiming to avoid disease progression and prevent patients at high risk from developing cardiovascular and cerebrovascular events.

In the current study, abnormal ABI was detected in 28% of the studied children with T1DM, of which 85.7% had ABI < 0.9 and 14.3% had ABI higher than 1.2. Nattero-Chávez and colleagues reported an overall prevalence of PAD of 12.8% among children with T1DM [29]. Another study including adults with T1DM described a prevalence of PAD of 27.7% [32]. The high prevalence of subclinical PAD among this population highlights the importance of addressing its genetic determinants.

Overall, children with T1DM had significantly lower ABI than controls. Low ABI is known to be a strong predictor of cardiovascular morbidity and mortality in people with diabetes [33]. In agreement with the current results, Pastore and colleagues reported increased subclinical vascular diseases among children with T1DM compared to healthy controls manifested as reduced brachial artery flow-mediated dilatation reactivity and increased carotid–femoral pulse wave velocity [34].

Although the relation between T1DM and PAD is well established, the determinants of PAD among children with T1DM remains unclear. The proposed predictors of PAD include glycemic and lipid derangements, diabetes duration and hypertension, and male gender [4]. In the current study, PAD was found to be correlated with diabetes duration, insulin dose, and lipid derangements; interestingly, it was not correlated to HbA1C. This goes in line with the DCCT/EDIC study that observed that lowering HbA1c is not solely enough to reduce the rate of PAD in those at risk [5].

In the context of adipokines gene polymorphism, children with T1DM were found to have significantly higher GG chemerin and TT adiponectin gene polymorphisms than controls. The pro-inflammatory chemerin and the anti-inflammatory adiponectin gene polymorphisms have been investigated separately in obesity and T2DM. However, data about their role in T1DM is scarce and lacking. Perumalsamy and coworkers found a significant association between chemerin gene polymorphism and insulin resistance and cardiovascular disease among adults with T2DM [35]. Moreover, the frequency of the G allele of the chemerin rs17173608 polymorphism was found to be significantly higher in people with T2DM than in controls [36]. Regarding adiponectin rs1501299 polymorphism, it has been linked significantly to T1DM in adults [37]. In addition, Cui and coworkers found a significant association between adiponectin rs1501299 polymorphism and T2DM among adults [38].

Interestingly, children with T1DM having GG chemerin variant and those having TT adiponectin variant were found to have significantly higher cholesterol and significantly lower HDL-C than those having the other two variants. Chemerin expression and secretion are reported to increase significantly with adipogenesis. A previous study showed a significant relation between LDL and GG chemerin genotype among adults with metabolic syndrome [39].

Moreover, chemerin was previously reported to have a strong positive correlation with triglycerides, cholesterol, and LDL with a strong negative correlation with HDL-C [40]. As for adiponectin, a previous study by Tsuzaki and coworkers found that HDL-C was significantly and independently correlated with adiponectin and adiponectin rs1501299 G allele by multivariate regression analysis [41].

In the present study, adiponectin and chemerin gene polymorphism was found to be independently associated with PAD among children with T1DM, which suggests a possible role for these polymorphisms in the development of PAD among this population. This goes in agreement with the study by Kennedy and coworkers which identified increased expression of chemerin in atherosclerotic coronary arteries, aorta, and mesenteric arteries [42]. They found that mRNA and protein of chemerin receptors are widely expressed in smooth muscles and endothelial cells of human vessels, reinforcing the importance of their role in vasculature modulation. Interestingly, the expression of chemerin they found in the vascular smooth muscles is similar to that reported for adiponectin [43]. Moreover, adiponectin gene mutations were found to be strongly associated with impaired glucose tolerance, diabetes mellitus, and coronary artery disease in humans [44]. In mice, a high-fat and high-sucrose diet was found to cause marked elevation of plasma glucose and insulin levels, insulin resistance, and an increase in intimal smooth muscle cell proliferation in adiponectin knockout mice [45].

## Conclusion

Subclinical PAD probably starts early in children with T1DM as denoted by abnormal ABI. This abnormal ABI is independently related to chemerin and adiponectin gene polymorphism.

Thus, chemerin and adiponectin gene polymorphisms could have a role in the pathogenesis of PAD among children with T1DM. Moreover, chemerin and adiponectin genes could be used as risk biomarkers for hyperlipidemia and vascular affection among children with T1DM.

## Limitations of the study

The cross sectional nature of the study could not prove causality and the relatively small sample size. Therefore, further larger longitudinal studies are needed to identify the role of chemerin and adiponectin gene polymorphisms in predicting cytokine levels and PAD risk among children with T1DM.

## Data Availability

Data will be available from the corresponding author upon request.

## Abbreviations

ABI: Ankle–brachial index
BMI: Body mass index
HDL-C: High-density lipoproteins
IQR: Inter-quartile range
LDL: Low-density lipoproteins
MODY: Maturity-onset diabetes of the youth
PAD: Peripheral artery disease
RARRES2: Retinoic acid receptor responder 2
ROC: Receiver operating characteristic
T1DM: Type 1 diabetes
T2DM: Type 2 diabetes
